# Evaluation of the compatibility from antibiotic use with the Gyssens plot in community acruired pneumonia patients in RSUP DR Wahidin Sudirohusodo

**DOI:** 10.1017/ash.2025.125

**Published:** 2025-09-03

**Authors:** Hasliyawati Hasan, Sudirman Katu, Risna Halim

**Affiliations:** Division of Tropical Infection, Department of Internal Medicine, Faculty of MedicineHasanuddin University

**Keywords:** antibiotic use, gyssens plot, community acquired pneumonia

## Abstract

**Objectives:** Pneumonia is the leading cause of morbidity and mortality worldwide, eighth rank in the United States. The use of antibiotic for pneumonia theraphy contribute the highest rate compared to the usage of antibiotic therapy for other diseases. Antibiotic resistance can occur due to the irrational use of antibiotics. World Health Organization (WHO) predicts that by 2050 there will be 10 million people per year who die due to drug resistance. The Gyssens method is a tool to evaluate the quality of antibiotic use that has been widely used in various countries. The purpose of this study was to assess the compatibility of antibiotic use based on Gyssens plot categories in pneumonia patients. **Methods:** This study used descriptive-observational with a cross-sectional study in pneumonia patients at Dr. Wahidin Sudirohusodo Hospital on periode from July-December 2023. The compatibility of antibiotics was assessed using the Gyssens flow. **Results:** This study involved 116 subjects, 70 males, 46 females. In this study, found that the use of rational antibiotics is 58% and irrational 42%. From irrational group we obtained category IV (3%), IIIa (31%), IIIb (8%). The most often antibiotic that used are ceftriaxone (26,8%)) and azithromycin (26,3%). **Conclusions:** The use of antibiotic in pneumonia patients assessed using the gyssens method in the Inpatient Installation of Dr. Wahidin Sudirohusodo Hospital from July to December 2023 found that the usage for antibiotic is rational for 58%, of the total patient and irrational use was 42% of the total patient during the research study.

